# Correlation between apparent diffusion coefficients and HER2 status in gastric cancers: pilot study

**DOI:** 10.1186/s12885-015-1726-7

**Published:** 2015-10-20

**Authors:** Jian He, Hua Shi, Zhuping Zhou, Jun Chen, Wenxian Guan, Hao Wang, Haiping Yu, Song Liu, Zhengyang Zhou, Xiaofeng Yang, Tian Liu

**Affiliations:** 1 Department of Radiology, Nanjing Drum Tower Hospital, The Affiliated Hospital of Nanjing University Medical School, Nanjing, 210008 China; 2 Department of Pathology, Nanjing Drum Tower Hospital, The Affiliated Hospital of Nanjing University Medical School, Nanjing, 210008 China; 3 Department of Gastrointestinal Surgery, Nanjing Drum Tower Hospital, The Affiliated Hospital of Nanjing University Medical School, Nanjing, 210008 China; 4 Radiation Oncology and Winship Cancer Institute, Emory University, Atlanta, GA 30322 USA

**Keywords:** Diffusion magnetic resonance imaging, Stomach neoplasms, Receptor, erbB-2, Immunohistochemistry, Molecular targeted therapy

## Abstract

**Background:**

To evaluate whether apparent diffusion coefficient (ADC) value of gastric cancer obtained from diffusion weighted imaging (DWI) correlates with the HER2 status.

**Methods:**

Forty-five patients, who had been diagnosed with gastric cancer through biopsy, were enrolled in this IRB-approved study. Each patient underwent a DWI (b values: 0 and 1,000 sec/mm^2^) prior to surgery (curative gastrectomy or palliative resection). Postoperative microscopic findings, HER2 status by immunohistochemical analysis and fluorescence in situ hybridization (FISH) were obtained. HER2 status was compared among gastric cancers with various histopathological features using the chi square test. The ADC values of gastric cancers with positive and negative HER2 were compared using the student t test.

**Results:**

A weak yet significant correlation was observed between the mean ADC values and HER2 status (r = 0.312, *P* = 0.037) and scores (r = 0.419, *P* = 0.004). The mean ADC value of HER2-positive gastric cancers was significantly higher than those of HER2-negative tumors (1.211 vs. 0.984 mm^2^/s, *P* = 0.020). The minimal ADC value of HER2-positive gastric cancers was significantly higher than those of HER2-negative tumors (1.105 vs. 0.905 × 10^−3^ mm^2^/s, *P* = 0.036).

**Conclusions:**

In this pilot study, we have demonstrated that the ADC values of gastric cancer correlate with the HER2 status. Future research is warranted to see if DWI can predict HER2 status and help in tailoring therapy for gastric cancer.

## Background

Gastric cancer is the second most common cause of cancer-related death worldwide [1]. When diagnosed at advanced stages, many gastric-cancer patients (especially at M1 or T4b stage) lose the opportunity of surgical resection, and chemotherapy is the most effective treatment to improve overall survival [2]. However, a meta-analysis indicated that neoadjuvant chemotherapy, with relative high adverse effects, doesn’t improve 3-year disease-free survival [3]. The use of trastuzumab, a monoclonal antibody against human epidermal growth factor receptor 2 (HER2; also known as ERBB2), plus chemotherapy proved to improve median overall survival in patients with advanced gastric or gastro-oesophageal junction cancer, compared with chemotherapy alone in ToGA trial [4]. Therefore, an accurate and reliable assessment of HER2 status is important for selecting patients with gastric cancer who may benefit from trastuzumab treatment [5]. However, current method assessing HER2 status either by using immunohistochemistry (IHC) or by fluorescence in situ hybridization (FISH) in tumor specimens obtained from surgical resection or endoscopic biopsy involves invasive procedures. Nowadays a biopsy is mandatory for the diagnosis and therefore the HER2 status can be known by IHC. However, care should be taken in approaching HER2 testing in the routine workflow for gastric cancer. [6].

Nowadays, Magnetic resonance (MR) imaging is increasingly used in diagnosis and staging of the gastric cancers because it is noninvasive and provides morphological as well as functional information [7]. In particular, the diffusion weighted imaging (DWI), which reflects the mobility of water molecule in vivo and can be quantified by apparent diffusion coefficient (ADC) values, has been widely investigated in various tumors [8–12]. Previous studies have demonstrated the value of DWI in detection [13] and characterization [14] of gastric cancers. MR imaging with DWI can increase the sensitivity and accuracy in TNM staging of gastric cancer [15, 16], especially in T staging [17, 18]. In quantitative terms, the ADC values could help to differentiate gastric cancers from normal gastric walls [13–15], gastric malignancies from benign diseases [19], and gastric adenocarcinoma from lymphoma [20]. The ADC values also seemed a useful tool to assess locally advanced gastric adenocarcinoma and gastro-oesophageal tumor response to neoadjuvant treatment [21, 22]. Additionally, the ADC value of metastatic nodes was reported significantly lower than that of the benign nodes [15, 23].

However, the correlation between the ADC values with HER2 status of gastric cancers remains unknown. Gastric cancers with different HER2 status have different structures and behaviors, which may be reflected by the ADC values [6, 24, 25]. Therefore, the purpose of this study was to explore the correlations between the ADC values and HER2 status of gastric cancers.

## Methods

### Patients

This study was approved by the ethics committee of Nanjing Drum Tower Hospital. The written informed consent was obtained from each patient. The inclusion criteria for patients were: 1) patients aged ≥ 18 years; 2) gastroscopic biopsy-confirmed histologic diagnosis of gastric carcinoma; 3) absence of any absolute contraindications to MR imaging (cardiac pacemaker or defibrillator, nerve stimulator, insulin pump, aneurysm clip, cochlear implant, etc.); 4) no prior local treatment or systematic chemotherapy of the gastric cancer; and 5) tumor thickness larger than 5 mm. Between December 2011 and March 2013, 45 consecutive patients (35 men and 10 women, age: 34 ~ 80 years; mean age: 60 years) were prospectively enrolled. Within 7 days of biopsy (range: 3 to 7 days; mean: 5 days) and prior to surgery, each patient underwent magnetic resonance imaging (MRI) in the Department of Radiology.

### MR examination

MR imaging was performed after the patients fasted for over 8 h to empty the gastrointestinal tract. After confirming no contraindications (glaucoma, prostate hypertrophy or severe heart disease) were presented for the patient, 20 mg of scopolamine butylbromide (1 ml: 20 mg; Chengdu NO.1 Drug Research Institute Company Limited, Chengdu, China) was injected intramuscularly to prevent gastrointestinal motility 10 min before MR imaging. The patients drank 800 to 1000 mL warm water 5 min before MR imaging to fill the gastric cavity. The patients were also trained to breathe normally before MR examinations.

MR imaging was performed using a clinical whole body 3.0 T scanner (Achieva 3.0 T TX; Philips Medical Systems, Best, the Netherlands) with a phased-array 16-channel sensitivity encoding multi-transmit abdominal coil. All patients were scanned in the head-first supine position. The field of view was set from the diaphragmatic dome to the level of the renal hilum.

MR sequences included: axial T2-weighted imaging, axial DWI and multiphase contrast enhanced T1 high resolution isotropic volume excitation imaging. Axial T2W images were obtained with respiratory-triggered turbo spin-echo sequence without fat-saturation (repetition time ms/echo time ms, 1210 ~ 1220/70; matrix, 256 × 198; section thickness 4 mm; gap, 1 mm; number of sections, 32 ~ 36; field of view, 36 cm; sensitivity encoding factor, 3.0; and number of signals averaged, 1). The scan time of T2W imaging was 1 min 36 s ~ 1 min 48 s. T1 high resolution isotropic volume excitation with spectral attenuated inversion recovery techniques (repetition time ms/echo time ms, shortest/shortest; matrix, 256 × 198; section thickness 4 mm; gap, 1 mm; number of sections, 32 ~ 36; field of view, 36 cm; and number of signals averaged, 1) were utilized before and 30, 60, 90 and 180 s after administration of 0.2 mL per kilogram of body weight gadodiamide (Omniscan 0.5 mmol/mL; GE Healthcare, Ireland) using an automatic power injector (Medrad Spectris Solaris EP MR Injector System; One Medrad Drive Indianola, PA, USA). The acquisition time of dynamic contrast enhanced MR imaging was 3 min 15 s ~ 3 min 17 s.

The axial DWI was performed using the respiratory-triggered single-shot spin-echo echo-planar sequence with chemical shift-selective fat-suppression techniques (b, 0 and 1000 sec/mm^2^; repetition time msec/echo time msec, 2280 ~ 3600/40 ~ 50; matrix, 236 × 186; section thickness, 4 mm; gap, 1 mm; field of view, 38 cm; number of sections, 32 ~ 36; number of signal averaged, 3). The diffusion weighted gradients were applied to the three orthogonal directions. The DWI scan time in this pilot study was 3 min 45 sec ~ 4 min 24 sec.

### Image analysis

Diffusion weighted images were analyzed in work station (Extended MR WorkSpace 2.6.3.4; Philips Medical Systems, Best, the Netherlands) and the ADC maps were generated by using a mono-exponential fit. All the MR images were carefully reviewed by two radiologists (Song Liu, Zhu Ping Zhou) with 5 to 6 years of experience in abdominal imaging. Both radiologists were informed with the location of the lesion, and were blind to the endoscopic and surgical pathological findings.

ADC map containing the largest slice of the tumor was adopted and one oval region-of-interest (ROI) was placed within the solid part of the lesion with consensus of two radiologists. The area of the ROIs (range: 20.3 ~ 95.2 mm^2^, mean: 45.7 mm^2^) may vary with the lesion size. If the lesion showed a sandwich sign [14], the ROI should avoid muscular layer. The mean and minimal ADC values of each ROI were recorded. The mean ADC value was defined as the arithmetic mean value of all the pixels within the ROI. The minimal ADC value was defined as the lowest value of all the pixels within the ROI. Additionally, the mean and minimal ADC values of normal gastric walls of all patients were also obtained. The area of ROIs for normal gastric wall (range: 21.5 ~ 56.9 mm^2^, mean: 37.5 mm^2^) varied with the location and distention status of the stomach.

### Surgical pathological analysis

Forty-two patients underwent total or partial curative gastrectomies; while 3 patients underwent palliative resections. Gastric specimens were analyzed by two pathologists with more than 10 years’ experience who were blinded to the MRI findings. The location, maximum diameter, histological type, differentiation degree (grade), Lauren classification and TNM stage of gastric cancer were evaluated.

A specific scoring system was introduced for the HER2 assessment of the gastric cancers, which was recently reinforced in consensus panel recommendations [6]. In detail, when considering HER2 protein status determination using IHC in gastric cancer resection, a patient was classified as score 3+ (IHC positive) if the membrane staining was strong complete, basolateral or lateral in >10 % of tumor cells; score 2+ (IHC equivocal) if the membrane staining was weak-to-moderate complete, basolateral or lateral in >10 % of tumor cells; score 1+ (IHC negative) if the membrane staining was faint/barely perceptible incomplete in >10 % of tumor cells; and score 0 (IHC negative) if no staining was observed or the membrane staining is in <10 % of tumor cells. Equivocal cases at IHC (2+ score) were subjected to fluorescence in situ hybridization (FISH) analysis. At a cytogenetic level, FISH interpretation criteria were based on a HER2/CEP17 ratio ≥2 as a cut off to define a HER2 FISH+ test.

### Statistical analysis

The mean and minimal ADC values of gastric cancers were compared among various HER2 statues as well as HER2 scores using a student t test or one-way variance analysis (F test). Correlations between mean and minimal ADC values of gastric cancers with various HER2 statues and scores were analyzed using the Spearman’s correlation test. HER2 status was compared among gastric cancers with various histological types, differential degrees, Lauren classifications and TNM stages using chi square test. The two operators reevaluated separately the whole DWI dataset and the interclass correlation coefficient (ICC) was estimated. The All statistical analyses were performed with SPSS 13.0 software (SPSS Inc, Chicago, Illinois). A P value less than 0.05 was considered statistically significant.

## Results

Each patient had one lesion identified. The tumors were located in the cardia and fundus (15 cases), cardia and body (4 cases), body (3 cases), body and antrum (4 cases) and antrum (19 cases) of the stomach. There were 24 poorly, 11 poorly to moderately, 8 moderately and 2 moderately to well differentiated carcinomas, respectively.

**Fig. 1:**
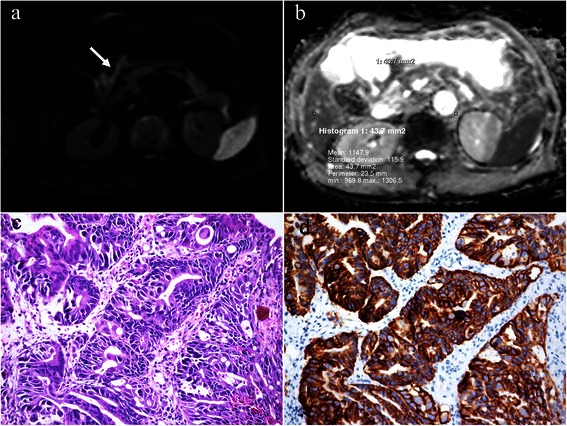
A 69-year-old man with gastric cancer, at stage IIIA (T3N2M0). Axial diffusion weighted image (b = 1000 sec/mm^2^) (**a**) shows the lesion with remarkably high signal intensity in antrum (*arrow*) of stomach with a maximum diameter of 5.0 cm. An oval region-of-interest (ROI) with an area of 43.7 mm^2^ is placed within the solid part of the lesion in corresponding apparent diffusion coefficient (ADC) map (**b**), which shows restricted mean and minimal ADC values as 1.148 and 0.970 × 10^−3^ mm^2^/s respectively. Photomicrograph (Hematoxylin & Eosin staining, ×200) (**c**) proves moderately differentiated adenocarcinoma with a Lauren classification of intestinal type. HER2 immunohistochemical assay (**d**) shows complete and intense circumferential membrane staining in >10 % of tumor cells (score 3+)

**Fig. 2:**
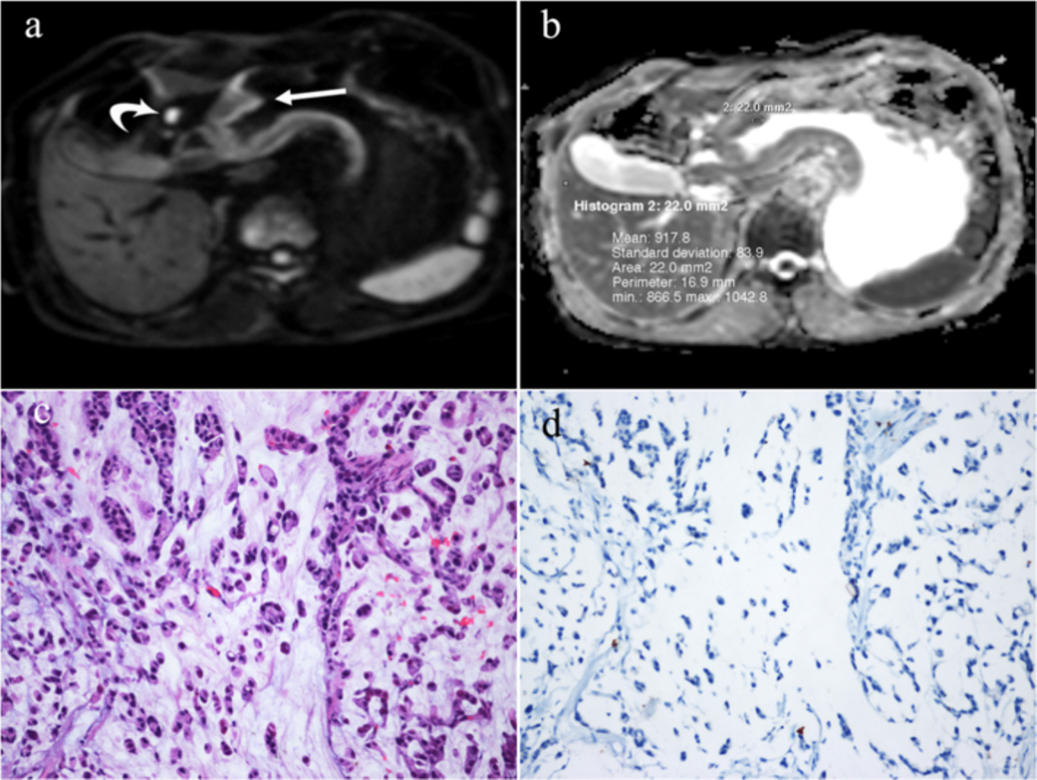
A 58-year-old man with gastric cancer, at stage III B (T3N3M0). Axial diffusion weighted image (b = 1000 sec/mm^2^) (**a**) shows a hyperintense lesion in antrum (*arrow*) of stomach with a maximum diameter of 6.0 cm. Note the bright lymph node metastasis (*curved arrow*). An oval region-of-interest (ROI) with an area of 22.0 mm^2^ is placed within the solid part of the lesion in corresponding apparent diffusion coefficient (ADC) map (**b**), which shows restricted mean and minimal ADC values as 0.918 and 0.867 × 10^−3^ mm^2^/s. Photomicrograph (Hematoxylin & Eosin staining, ×200) (**c**) reveals signet ring cell carcinoma with a Lauren classification of diffuse type. HER2 immunohistochemical assay (**d**) shows no membrane staining is observed (score 0)

Among the 45 cases, HER2 antibody measured by IHC staining was 3+ in 5 (11.1 %), 2+ in 7 (15.6 %) with, 1+ in 9 (20.0 %), and negative in 24 (53.3 %). FISH was positive in 4 out of the 7 IHC 2+ cases. Hence, there were total 9 (20.0 %) positive HER2 cases (Fig. 1) and 36 (80.0 %) negative HER2 cases (Fig. 2).

**Fig. 3:**
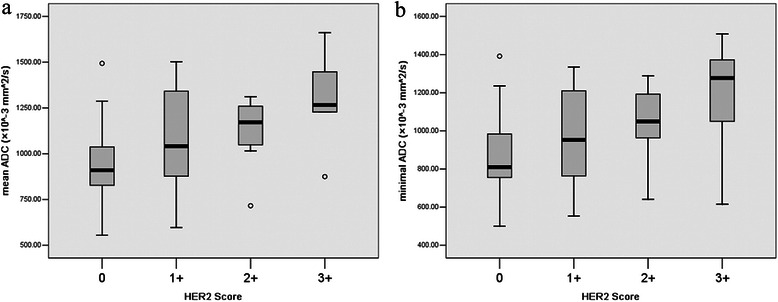
Box plots of mean (**a**) and minimal (**b**) apparent diffusion coefficient (ADC) values of gastric cancers with different HER2 scores

The mean and minimal ADC values of the gastric cancers with various HER2 statuses were shown in Table 1. A weak, yet significant correlation was observed between mean ADC values of the gastric cancers and HER2 status (*r* = 0.312, *P* = 0.037), and between the mean of ADC values and HER2 scores (*r* = 0.419, *P* = 0.004) (Fig. 3). The minimal ADC values of gastric cancers correlated with HER2 scores (*r* = 0.367, *P* = 0.013) rather than HER2 status (*r* = 0.282, *P* = 0.060).

**Table 1:** Mean and min ADC values (×10^−3^ mm^2^/s) of gastric cancers with different HER2 status

HER2 status	n	mean ADC	*P* value	min ADC	*P* value
Score (0)	24	0.934 ± 0.215	0.022^*^	0.861 ± 0.217	0.060^*^
Score (1+)	9	1.068 ± 0.312		0.970 ± 0.273	
Score (2+)	7	1.116 ± 0.208		1.041 ± 0.217	
Score (3+)	5	1.295 ± 0.291	0.004^§^	1.164 ± 0.349	0.016^§^
Negative (-)	36	0.984 ± 0.242	0.020^‡^	0.905 ± 0.230	0.036^‡^
Positive (+)	9	1.211 ± 0.286		1.105 ± 0.314	

*one-way analysis of variance among score 0-3; §LSD method between score 0 and 3+; ‡student t test between negative (-) and positive (+) groups

The mean and minimal ADC values of HER2-positive gastric cancers were significantly higher than those of HER2-negative tumors (1.211 vs. 0.984, 1.105 vs. 0.905 × 10^−3^ mm^2^/s, *P* = 0.020, 0.036, respectively). The mean and minimal ADC values of HER2 (3+) gastric cancers were significantly higher than those of HER2-negative (0) tumors (1.295 ± 0.290 vs. 0.934 ± 0.215, 1.164 ± 0.349 vs. 0.861 ± 0.217 × 10^−3^ mm^2^/s, *P* = 0.004, 0.016, respectively).

The histopathological features of the gastric cancers with various HER2 statuses were shown in Table 2. The HER2-positive rate in gastric cancers of the interstitial type (6/11, 54.5 %) was significantly higher than that of the diffuse type (2/30, 6.7 %) (*P* = 0.003).

**Table 2:** Histopathological features in gastric cancers with different HER2 status

	HER2(+)	HER2(-)	*P* value
N	9	36	
Gender (M/F)	6/3	23/13	0.876
Age (mean ± SD)	60.3 ± 10.7	61.6 ± 11.2	0.755
Diameter (mean ± SD)	5.11 ± 2.89	5.11 ± 2.21	1.000
Diameter (<3 cm/>3 cm)	2/7	10/27	0.768
Diameter (<4 cm/>4 cm)	5/4	14/22	0.365
T stage (1/2/3/4)	0/2/6/1	6/5/20/5	0.569
N stage (0/1-3)	2/7	9/27	0.862
M stage (0/1)	8/1	35/1	0.278
TNM stage (I/II/III/IV)	1/2/5/1	6/11/18/1	0.686
Lauren classification (intestinal/ mixed/diffuse type)	6/1/2	5/3/28	0.003^*^
Pathologic type(adenocarcinoma /signet ring cell cancer)	7/2	30/6	0.697
Differentiation degree of adenocarcinomas (poor/poor-mod/mod/mod-well)^§^	4/2/1/0	12/9/7/2	0.784
Overall differentiation degree (poor/poor-mod/mod/mod-well)^‡^	6/2/1/0	18/9/7/2	0.758
Location (cardia&fundus/cardia&body/ body/body&antrum/antrum)	2/2/0/2/3	13/2/3/2/16	0.209

§poor, poorly; poor-mod, poorly to moderately; mod, moderately; mod-well, moderately to well; ‡ signet ring cell cancer was treated as poorly differentiated; * *P *< 0.05

The ICC of mean ADCs between two operators was 0.987 (95 % confidence interval: 0.982 ~ 0.990, *P* < 0.001) and the ICC of minimal ADC was 0.954 (95 % confidence interval: 0.929 ~ 0.969, *P* < 0.001), which showed an excellent inter-reader agreement of measured ADC values.

## Discussion

The HER2 (also known as ErbB2, c-erbB2, or Her2/neu) gene is located on chromosome 17q and encodes a 185 kDa transmembrane tyrosine kinase receptor protein with no known ligand [23]. HER2 forms both homo- and heterodimers and leads to activation of downstream signaling pathways to promote cell proliferation and suppress apoptosis, which may facilitate excessive/uncontrolled cell growth and tumorigenesis [26]. HER2 overexpression and/or amplification was reported in various solid tumors, such as breast, gastric, ovarian [27, 28], colorectal [29], salivary gland [30], bladder [31], and lung cancers [32]. The importance of HER2 as a key marker in gastric tumorigenesis has very recently come to light. Because of differences in the examination method and objective criteria, the frequency of HER2-positive gastric cancer varies considerably between studies, ranging from 6.0 % to 29.5 % in earlier studies [33]. A number of studies have shown that HER2 overexpression and amplification are related to the Lauren histological classification, with higher HER2 positivity rate found in the intestinal phenotype than in diffuse and mixed types [34], which was consistent with our finding. Recent study shows that HER2 alteration or overexpression was more frequently observed in the well or moderately differentiated type than poorly-differentiated gastric cancers [35–37]. Her2 expression/amplification was also associated with earlier tumor stages and absence of lymph node metastases [38]. Other factors correlating with HER2 overexpression include age, gender, tumor location, size, histological type, Bormann type, et al [39]. However, the prognostic value of HER2 amplification/over-expression in patients with gastric cancer remains controversial [24, 40].

We found that the ADC values of gastric cancers were higher in patients with positive HER2 expression than negative neoplasms. Our previous studies have confirmed the correlations between ADC values of gastric cancers with the Lauren classifications, differential degrees and TNM stages [41]. We found that ADC values of gastric cancer with intestinal type, well differentiation and early stages were higher than those with diffuse type, poor differentiation and advanced stages. Tubular or gland structures are commonly observed in the intestinal type, which may lead to relatively large spaces for water molecular Brownian motion. Low differentiation degree and high level of cellular atypia are common features of diffuse type, which may cause narrower and more distorted intercellular spaces. We also found that the ADC values of the gastric cancer correlated inversely with T stage [41]. We hypothesized that as the T stage improves the amount and density of tumor cells increase while their arrangement is disordered. Large cell column, increased nucleus/cytoplasm ratio and irregular cell shape cause narrower and distorted intercellular spaces, and as a result, a decreased ADC value. Since HER2 expression is higher in gastric cancers with intestinal type, well differentiation and early stages, and consequently, a higher ADC values were observed in gastric cancers with positive HER2 expression.

Similar correlation between ADC values and HER2 status can also been observed in breast cancers. Ji Hyun Youk et al. [42] reported that the mean ADC value of triple-negative invasive breast cancer was significantly higher than that of ER+ (*P* = 0.002) and HER2+. However, Melania Costantinithe et al. [43] found that average ADC values measured in triple-negative breast cancer were slightly lower than those observed in HER2-overexpressing subgroups with no statistical significance. Bo Bae Choi et al. [44] found significant low ADC values in invasive ductal carcinoma with HER2-negative expression (*P* <0.05). And Laura Martincich et al. [45] found that the subtype of pure HER2-enriched tumors had the highest median ADC value (1.190 × 10^−3^ mm^2^/s), compared with the other immunohistochemically defined intrinsic tumor subtypes.

As the overexpression and/or amplification of HER2 enables the constitutive activation of growth signaling pathways which are heavily involved in the carcinogenetic process, direct targeting of HER2 and inhibition of the HER2-activated signal transduction is likely to provide therapeutic possibilities for HER2-positive patients [46]. A recent meta-analysis has confirmed that addition of trastuzumab (anti-HER2 antibody) to chemotherapy for gastric and gastroesophageal cancer significantly improved outcome of overall survival and progression-free survival endpoints, while other monoclonal antibodies led to no improvement in results [25]. Trastuzumab in combination with chemotherapy is currently routine practice for patients with HER2-positive advanced esophagogastric adenocarcinoma [47].

DWI has been widely applied to predict and monitor treatment response in different types of neoplasm [48–50]. Francesco DC et al. [21] reported that the pretreatment ADC values of gastro-oesophageal cancer in responders were significantly lower and increased significantly after neoadjuvant treatment of radio-chemotherapy, since high pre-treatment ADC values are related to the presence of necrotic components, poor perfusion, and hypoxic environment, leading to a reduced sensitivity to neoadjuvant treatment. Therefore, DWI shows the potential to identify pre-treatment features affecting the gastric cancer response to neoadjuvant treatment. However, Francesco G et al. [22] reported that no significant differences could be found for prechemotherapy ADC values of gastric adenocarcinoma between responders and nonresponders. Anyway, no study on the performance of ADC values in trastuzumab plus chemotherapy for gastric cancers has been reported, which needs further studies.

There were some limitations in our study. Firstly, the samples size was relatively small and the correlation between ADC values and HER2 status was weak. Since the application of DWI in gastric cancer is just beginning, it was relatively large in this field and is essentially enough for a pilot study. We are accumulating more cases to confirm our findings in this study. Secondly, the pathologic foundation and mechanism of higher ADC values in gastric cancers with positive HER2 expression were only speculative. Thirdly, other biomarkers such as epidermal growth factor receptor 1 and 3, vascular endothelial growth factor, Ki67 were not analyzed in this study. Fourthly, b = 0 and 1000 sec/mm^2^ cannot eliminate the influence of perfusion, which could be resolved by using multiple b values with bi-exponential model. Further studies are required to address those limitations.

## Conclusions

ADC values from DWI reflected different HER2 status of gastric cancers. Although the molecular biomarkers of gastric cancers cannot be determined solely by ADC values, DWI procedure is noninvasive, costs short acquisition and post-processing time, and requires no contrast agent administration. Preoperative DWI, which provides real quantitative functional parameter, is promising in tailoring therapy for gastric cancers.

## Abbreviations

ADC: Apparent diffusion coefficient
DWI: Diffusion weighted imaging
FISH: Fluorescence in situ hybridization
HER2: Human epidermal growth factor receptor 2
ICC: Interclass correlation coefficient
IHC: Immunohistochemistry
MR: Magnetic resonance
MRI: Magnetic resonance imaging
ROI: Region-of-interest
